# The impact of DPP-4 inhibitors on long-term survival among diabetic patients after first acute myocardial infarction

**DOI:** 10.1186/s12933-017-0572-0

**Published:** 2017-07-11

**Authors:** Mei-Tzu Wang, Sheng-Che Lin, Pei-Ling Tang, Wang-Ting Hung, Chin-Chang Cheng, Jin-Shiou Yang, Hong-Tai Chang, Chun-Peng Liu, Guang-Yuan Mar, Wei-Chun Huang

**Affiliations:** 1 Critical Care Center and Cardiovascular Medical Center, Kaohsiung Veterans General Hospital, Kaohsiung, Taiwan; 2 School of Medicine, National Yang-Ming University, Taipei, Taiwan; 3 Department of Physical Therapy, Fooyin University, Kaohsiung, Taiwan; 4 Section of Critical Care Medicine, Kaohsiung Veterans General Hospital, No. 386, Dazhong 1st Rd., Zuoying Dist., Kaohsiung City, 813 Taiwan

**Keywords:** DPP-4 inhibitor, Acute myocardial infarction, Diabetes

## Abstract

**Background:**

Previous studies regarding the cardioprotective effects of dipeptidyl peptidase 4 (DPP-4) inhibitors have not provided sufficient evidence of a relationship between DPP-4 inhibition and actual cardiovascular outcomes. This study aimed to evaluate the impact of DPP-4 inhibitors on the survival of diabetic patients after first acute myocardial infarction (AMI).

**Methods:**

This was a nationwide, propensity score-matched, case–control study of 186,112 first AMI patients, 72,924 of whom had diabetes. A propensity score, one-to-one matching technique was used to match 2672 controls to 2672 patients in the DPP-4 inhibitor group for analysis. Controls were matched based on gender, age, and a history of hypertension, dyslipidemia, diabetes, peripheral vascular disease, heart failure, cerebrovascular accident, end-stage renal disease, chronic obstructive pulmonary disease, and percutaneous coronary intervention.

**Results:**

DPP-4 inhibitors improve the overall 3-year survival rate (log rank P < 0.0001), whether male or female. Cox proportional hazard regression showed DPP-4 inhibitor is beneficial in diabetes patients after AMI (HR = 0.86; 95% CI 0.78–0.95), especially in those patients with hypertension (HR = 0.87; 95% CI 0.78–0.97; P = 0.0103) and cerebrovascular disease (HR = 0.83; 95% CI 0.72–0.97; P = 0.018), but without dyslipidemia (HR = 0.78; 95% CI 0.67–0.92; P = 0.0029), without peripheral vascular disease (HR = 0.86; 95% CI 0.78–0.96; P = 0.0047), without heart failure (HR = 0.84; 95% CI 0.73–0.96; P = 0.0106), without end stage renal disease (HR = 0.86; 95% CI 0.77–0.95; P = 0.0035), and without chronic obstructive pulmonary disease (HR = 0.87; 95% CI 0.78–0.97; P = 0.0096).

**Conclusions:**

DPP-4 inhibitor therapy improved long-term survival in diabetic patients after first AMI, regardless of gender.

## Introduction

Diabetes mellitus (DM) with hyperglycemia and insulin resistance is one of the main risk factors for cardiovascular disease. Dipeptidyl peptidase 4 inhibitors (DPP-4i) are one of oral hypoglycemic agents (OHA) commonly used in type 2 DM patients. The effects of DPP-4i are mediated through the incretin hormones, glucagon-like peptide 1 (GLP-1) and gastric inhibitory peptide, by slowing gastric emptying, stimulating glucose-dependent insulin release from the pancreatic islets, and inhibiting inappropriate post-meal glucagon release [1].

In several animal studies, DPP-4 inhibitors were shown to achieve cardioprotective effects via several mechanisms. These effects included a reduction in reperfusion injury, an increase in threshold of ventricular fibrillation during the ischemic period, an induced antiapoptotic effect, a reduction in oxidative stress, a decrease in infarct size, stabilized cardiac electrophysiology, protected cardiac mitochondrial function, an inhibition of atherosclerosis, and vascular smooth muscle cell proliferation [2–7].

In human randomized, double-blind study studies, DPP-4i did not appear to increase the risk of major adverse cardiovascular events among patients with type 2 DM with established cardiovascular disease [8, 9]. However, definitive proof of an effect of DPP-4i in patients with acute coronary syndrome (ACS) is currently lacking. In a randomized trial, DPP-4i were shown to have a neutral effect on rates of major adverse ischemic cardiovascular events and to have increased the rate of hospitalization for heart failure in DM patients with ACS [10, 11]. Whereas, other prospective, open-labeled, randomized studies showed that DPP-4i improved coronary flow reserve and left ventricular election fraction, and achieved regression of coronary artery plaque or reduction in major cardiovascular events [12, 13].

This study aimed to evaluate the impact of DPP-4i on survival of diabetic patients after first acute myocardial infarction (AMI) through analysis of the data from the Taiwan National Health Insurance Research Database.

## Methods

### Data source

Since 1995, the National Health Insurance Program has provided healthcare to approximately 99% of the residents in Taiwan. The data for this study were collected from National Health Insurance Research Database (NHIRD) from January 2000 through December 2012.

The NHIRD includes detailed information from medical inpatient records including age, gender, diagnosis, interventional procedures, medical orders, and relevant survival data. The database provides researchers with de-identified data via encryption of the identification codes to preserve patient anonymity and has been extensively used in epidemiologic studies in Taiwan. This study was approved by the Human Research Committee of Kaohsiung Veterans General Hospital.

### Definition of AMI population

**Fig. 1:**
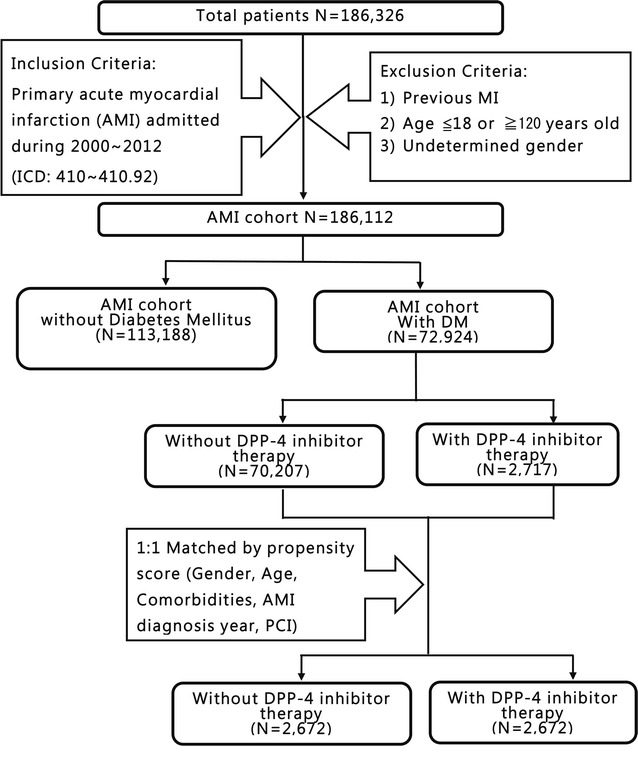
Flowchart outlining the various study cohorts. There were 186,326 patients in Taiwan between January 2000 and December 2012 with a primary diagnosis of acute myocardial infarction (AMI) (ICD codes: 410–410.92). From this group, patients were excluded who had a previous admission for AMI, who were ≤18 or ≥120 years old, and whose gender was undetermined. Among the remaining 186,112 cases with a primary diagnosis AMI, 72,924 cases had diabetes mellitus and underwent propensity score matching to controls to minimize baseline differences between the two groups. 2672 AMI patients with DPP-4i and 2672 matched controls were, therefore, included in our final analysis. *AMI* acute myocardial infarction, *DM* diabetes mellitus, *DPP*-*4* dipeptidyl peptidase 4, *PCI* percutaneous coronary intervention

A total of 186,326 patients admitted to hospitals in Taiwan between January 2000 and December 2012 with a primary diagnosis of AMI (ICD: 410–410.92) were retrieved from Taiwan’s NHIRD which consisted of data collected from more than 23,000,000 patients. From this group of 186,326 patients, those with a previous admission for AMI, those who were ≤18 or ≥120 years old, and those patients whose gender was undetermined were excluded. The remaining 186,112 patients were included in the analysis (Fig. 1).

### Study population

Among the 186,112 patients who were hospitalized for first AMI, 72,924 cases with DM were identified. The remaining 113,188 patients without DM were excluded. A propensity score matching technique was applied to minimize baseline differences between the control group and the DPP-4i group. One-to-one matching was performed using the following variables: gender, age, hypertension, dyslipidemia, diabetes, HF, peripheral vascular disease (PVD), cerebrovascular accident (CVA), end-stage renal disease (ESRD), chronic obstructive pulmonary disease (COPD), and percutaneous coronary intervention (PCI) (Table 1). The data from 2672 AMI patients receiving DPP-4i and 2672 matched controls were included in the final analysis (Fig. 1).

**Table 1:** Patient characteristics first hospitalized for AMI with and without DPP-4 inhibitor

Characteristics	Control group (N = 2672)	DPP-4 inhibitor group (N = 2672)	P value
Gender			
Female	1098 (41.09%)	1098 (41.09%)	1
Male	1574 (58.91%)	1574 (58.91%)	
Age			
Age <65	1064 (39.82%)	1059 (39.63%)	0.8468
65 ≤ age < 75	707 (26.46%)	725 (27.13%)	
Age ≥75	901 (33.72%)	888 (33.23%)	
Comorbidities			
Hypertension	2016 (75.45%)	2005 (75.04%)	0.7274
Dyslipidemia	1996 (74.7%)	1996 (74.7%)	1
Peripheral vascular disease	144 (5.39%)	144 (5.39%)	1
Heart failure	971 (36.34%)	994 (37.2%)	0.5141
End stage renal disease	187 (7%)	187 (7%)	1
COPD	231 (8.65%)	234 (8.76%)	0.8842
Cerebrovascular accident	789 (29.53%)	796 (29.79%)	0.8339
Operations			
Percutaneous coronary intervention	1699 (63.59%)	1699 (63.59%)	1
Coronary artery bypass graft	260 (9.73%)	297 (11.12%)	0.0976
IABP	240 (8.98%)	248 (9.28%)	0.704
Medication			
Aspirin	2330 (87.2%)	2440 (91.32%)	<0.0001
Clopidogrel	2350 (87.95%)	2454 (91.84%)	<0.0001
Any anti-platelet drug	2513 (94.05%)	2596 (97.16%)	<0.0001
ACEI or ARB	1743 (65.23%)	2007 (75.11%)	<0.0001
Statin	1616 (60.48%)	1780 (66.62%)	<0.0001
β-Blocker	1587 (59.39%)	1884 (70.51%)	<0.0001
CCB	980 (36.68%)	1145 (42.85%)	<0.0001
Heparin or low molecular weight heparin	2174 (81.36%)	2266 (84.81%)	0.0008
Spironolactone	468 (17.51%)	615 (23.02%)	<0.0001
Nitrate	2245 (84.02%)	2366 (88.55%)	<0.0001
Nicorandil	284 (10.63%)	401 (15.01%)	<0.0001
α-Glucosidase	197 (7.37%)	602 (22.53%)	<0.0001
Glinides	311 (11.64%)	644 (24.1%)	<0.0001
Metformin	781 (29.23%)	1168 (43.71%)	<0.0001
Sulfonylureas	778 (29.12%)	1420 (53.14%)	<0.0001

*ACEI* angiotensin-converting enzyme inhibitor, *ARB* angiotensin receptor blocker, *CCB* calcium channel blocker, *COPD* chronic obstructive pulmonary disease

### Outcome analysis

Survival was defined based on the difference between the date of hospitalization and the end date of National Health Insurance (NHI) coverage. Since the NHI premium is paid monthly, coverage can easily be canceled at the time of death. Measurement of mortality was valid via the record of the end date of NHI coverage, within a maximum error of 1 month [14, 15].

### Statistical analysis

The SAS version 9.4 software (SAS Institute, Inc., Cary, NC) was used to analyze the data in this study. All variables were calculated using descriptive statistics. Categorical data were expressed as percentile values and continuous variables were expressed as a mean and standard deviation (SD). Paired t test for continuous variables and Chi squared test for categorical variables were applied to evaluate between-group differences. A P < 0.05 was considered statistically significant.

Cox proportional hazard regression analysis was used to calculate the hazard ratio (HR) and the associated 95% confidence intervals (95% CIs) for significant variables. Kaplan–Meier cumulative survival curves were used to compare survival between patients who received DPP-4i compared with those who did not receive DPP-4i in order to compare survival between the two groups as a whole, based on gender, and also based on age. Log-rank tests which used a P < 0.05 were considered statistically significant.

## Results

The descriptive characteristics of the 2672 patients in the AMI patient group with diabetes who also received DPP-4i (the DPP-4i group) and the 2672 matched controls (the control group) are listed in Table 1. The two groups were comparable with regards to age, gender, comorbidities, and number and type of surgery. However, the patients in the DPP-4i group exhibited a higher use of anti-platelet drugs, angiotensin-converting enzyme inhibitors (ACEIs), angiotensin receptor blockers (ARBs), calcium channel blockers (CCBs), β-blockers, heparin, low molecular weight heparin, spironolactone, nitrates, and nicorandil (Table 1). Furthermore, the patients in DPP-4i also received a greater proportion of the other classes of oral hypoglycemic agents compared with controls (Table 1).

**Fig. 2:**
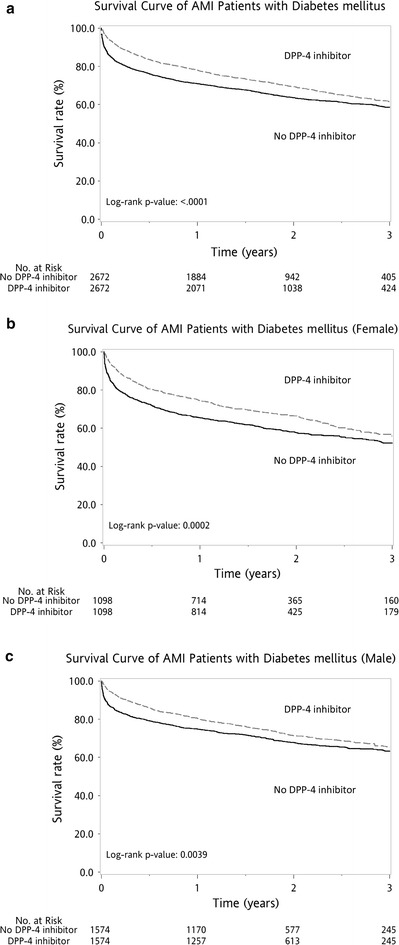
Kaplan–Meier survival curve after first acute myocardial infarction (AMI) for gender subgroup analysis. Overall, the 3-year survival rate was higher for the DPP-4i group than for the control group (log-rank P < 0.0001, **a**), regardless of gender [female (**b**) or male subgroup (**c**)]

Overall, the 3-year survival rate was significantly higher in the DPP-4i group when compared with the control group (log-rank P < 0.0001), regardless of gender (log-rank P = 0.0039 for males and log-rank P = 0.0002 for females) (Fig. 2).

**Fig. 3:**
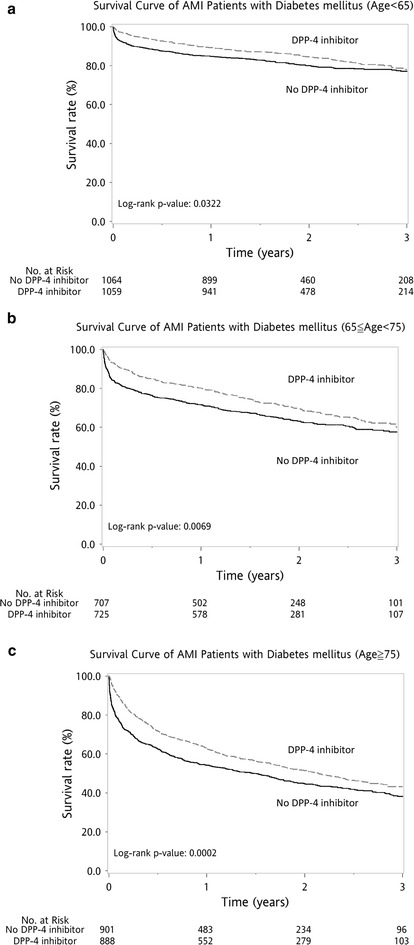
Kaplan–Meier survival curve after first acute myocardial infarction (AMI) for age subgroup analysis. Patients in the DPP-4i and control groups were subdivided into three subgroups by age. Kaplan–Meier cumulative survival curves revealed better survival in all three subgroups [age <65 years (log-rank P = 0.0322, **a**), 65 ≤ age < 75 years (log-rank P = 0.0069, **b**), and age ≥75 (log-rank P = 0.0002, **c**)]

The patients were divided into three subgroups based on age. The Kaplan–Meier cumulative survival curves revealed better survival in the DPP-4i group among all three age subgroups, i.e., age <65 years (log-rank P = 0.0322), 65 ≤ age < 75 years (log-rank P = 0.0069), and age ≥75 (log-rank P = 0.0002) (Fig. 3).

Cox proportional hazard regression analysis was performed to further evaluate the impact of DPP-4i on the survival of DM patients after first AMI (Table 2). Overall, HRs for mortality were higher in patients who were relatively older, i.e., 65 ≤ age < 75 compared with age <65 (HR = 1.71; 95% CI 1.5–1.96) and age ≥75 compared with age <65 (HR = 2.51; 95% CI 2.2–2.85). In addition, mortality was higher in patients with hypertension (HR = 1.2; 95% CI 1.06–1.37), peripheral vascular disease (HR = 1.57; 95% CI 1.34–1.84), heart failure (HR = 1.35; 95% CI 1.23–1.49), ESRD (HR = 1.76; 95% CI 1.51–2.06), previous stroke (HR = 1.33; 95% CI 1.21–1.47), and COPD (HR = 1.33; 95% CI 1.16–1.52). PCI was shown to reduce the risk of mortality in DM patients after AMI (HR = 0.54; 95% CI 0.49–0.60).

**Table 2:** Cox proportional hazard regression analysis on mortality of diabetic patients after first acute myocardial infarction

Characteristics (all, N = 5344)	HR (95% CI)
Sex (male vs female)	0.98 (0.89–1.07)
Age (65 ≤ age < 75 vs age <65)	1.71 (1.5–1.96)***
Age (age ≥75 vs age <65)	2.51 (2.2–2.85)***
Hypertension (yes vs no)	1.2 (1.06–1.37)**
Peripheral vascular disease (yes vs no)	1.57 (1.34–1.84)***
Heart failure (yes vs no)	1.35 (1.23–1.49)***
End stage renal disease (yes vs no)	1.76 (1.51–2.06)***
Cerebrovascular disease (yes vs no)	1.33 (1.21–1.47)***
Chronic obstructive pulmonary disease (yes vs no)	1.33 (1.16–1.52)***
Percutaneous coronary intervention (yes vs no)	0.54 (0.49–0.6)***
Any antiplatelet (yes vs no)	0.58 (0.49–0.7)***
ACEI or ARB (yes vs no)	0.72 (0.65–0.8)***
β-Blocker (yes vs no)	0.79 (0.71–0.87)***
Heparin or low molecular weight heparin (yes vs no)	1.02 (0.91–1.15)
α-Glucosidase (yes vs no)	0.95 (0.83–1.08)
Glinides (yes vs no)	1.05 (0.94–1.18)
Metformin (yes vs no)	0.77 (0.68–0.86)***
Sulfonylureas (yes vs no)	0.91 (0.82–1.01)
Thiazolidinedione (yes vs no)	0.79 (0.59–1.04)
DPP-4 inhibitor (yes vs no)	0.86 (0.78–0.95)**

** P < 0.01, *** P < 0.001

Regarding medications, DPP-4i therapy improved overall survival (HR = 0.86; 95% CI 0.78–0.95), and metformin also made contributions to overall survival (HR = 0.77; 95% CI 0.68–0.86). Other medications improved survival, including β-blockers (HR = 0.79; 95% CI 0.71–0.87), anti-platelet drugs (HR = 0.58; 95% CI 0.49–0.70), and ACEIs or ARBs (HR = 0.72; 95% CI 0.65–0.80) (Table 2).

**Fig. 4:**
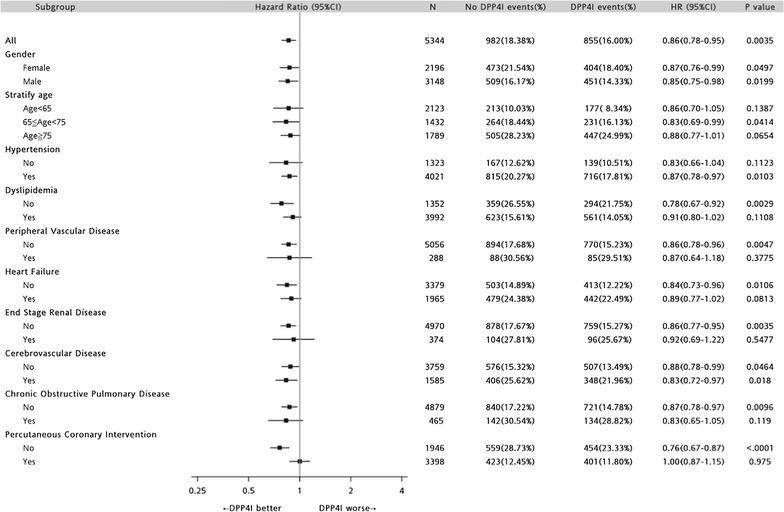
Forest plot of hazard ratios for various patient characteristics with or without DPP-4 inhibitor (DPP-4i). DPP-4 inhibitors therapy is beneficial in both male and female patients. DPP-4i therapy is beneficial in patients who are 65 ≤ age < 75 (HR = 0.83; 95% CI 0.69–0.99; P = 0.0414). In addition, a positive effect of DPP-4i can be seen in patients without dyslipidemia (HR = 0.78; 95% CI 0.67–0.92; P = 0.0029), without peripheral vascular disease (PVD) (HR = 0.86; 95% CI 0.78–0.96; P = 0.0047), without heart failure (HF) (HR = 0.84; 95% CI 0.73–0.96; P = 0.0106), without end stage renal disease (ESRD) (HR = 0.86; 95% CI 0.77–0.95; P = 0.0035), and those without chronic obstructive pulmonary disease (COPD) (HR = 0.87; 95% CI 0.78–0.97; P = 0.0096). DPP-4i are also beneficial in patients with hypertension (HR = 0.87; 95% CI 0.78–0.97; P = 0.0103) and cerebrovascular disease (HR = 0.83; 95% CI 0.72–0.97; P = 0.018)

DPP-4 inhibitors therapy is beneficial in both male and female patients (Fig. 4). In addition, a Forest plot of HRs for various patient characteristics with or without DPP-4i revealed that DPP-4i had better outcomes in patients with hypertension (HR = 0.87; 95% CI 0.78–0.97; P = 0.0103) and cerebrovascular disease (HR = 0.83; 95% CI 0.72–0.97; P = 0.018), but without dyslipidemia(HR = 0.78; 95% CI 0.67–0.92; P = 0.0029), without peripheral vascular disease (HR = 0.86; 95% CI 0.78–0.96; P = 0.0047), without heart failure (HR = 0.84; 95% CI 0.73–0.96; P = 0.0106), without end stage renal disease (HR = 0.86; 95% CI 0.77–0.95; P = 0.0035), and without chronic obstructive pulmonary disease (HR = 0.87; 95% CI 0.78–0.97; P = 0.0096) (Fig. 4).

## Discussion

This study demonstrated that use of DPP-4i in AMI patients with diabetes resulted in improved 3-year survival rates. DPP-4i therapy was especially beneficial in hypertension and cerebrovascular disease, regardless of gender, and in patients without peripheral vascular disease, end stage renal disease and chronic obstructive pulmonary disease.

### The impact of DPP-4 inhibitors on cardiovascular disease

Most of the prior literature has focused on the relationship between DPP-4i and cardiovascular safety. Several recent studies (including pre-clinical data, small mechanistic studies, and post hoc analyses of randomized clinical trials) support the benefit of DPP-4i in patients with cardiovascular disease [16–18]. In a nationwide longitudinal study, DPP4i-treated T2DM patients were shown to have a lower risk for cardiovascular disease as compared with non-DPP4i users [19]. Furthermore, in DM patients with pre-existing heart failure, the use of DPP-4i has resulted in a lower risk of mortality in patients with the combination of myocardial infarction and ischemic stroke [16]. Some studies have discussed the effect of DPP-4i on cardiovascular outcomes, but were limited regarding their ability to evaluate the impact of DPP-4i on the long-term outcomes after first AMI.

### The impact of DPP-4 inhibitors on DM patients after AMI

In a murine experimental diabetes model, DPP-4 inhibition was shown to attenuate cardiac dysfunction and adverse remodeling in the post-MI setting [20]. Furthermore, chronic treatment with DPP-4i in an animal model reduced infarct size and improved LV function, via the GLP-1 receptor-protein kinase A pathway, in a glucose-dependent manner [21, 22]. Peak plasma troponin I did not differ between patients with myocardial infarction on DPP4i and those who did not receive such therapy [23]. Rather than a neutral or negative effect, our study provides evidence that DPP-4i therapy can increase the survival of patients following a first AMI regardless of gender. In this study, DPP-4i therapy after first AMI improved long-term survival rather than resulting in just a “do no harm” effect. Major prospective clinical trials are investigating the various uses and cardiovascular outcomes of DPP-4i in diabetic patients. These trials include the Sitagliptin Cardiovascular Outcome Study (TECOS), the saxagliptin assessment of vascular outcomes recorded in patients with diabetes mellitus-thrombolysis in myocardial infarction (SAVOR-TIMI), the cardiovascular outcomes study of alogliptin in subjects with type 2 diabetes and acute coronary syndrome (EXAMINE), and the cardiovascular outcome study of linagliptin versus glimepiride in patients with type 2 diabetes (CAROLINA) trial [24–26]. It would be an important milestone and significant influence on the management of diabetes if these trials confirm that DPP-4i can contribute to a reduction in the cardiovascular complications of DM.

### The mechanisms underlying the DPP-4 inhibitor benefit

There are several possible explanations for the benefit of DPP-4i in patients with AMI. First, DPP4i may reduce reperfusion injury via protection of mitochondrial function [27]. DPP-4i has been shown to elevate the activity of reperfusion injury salvage kinase and reduce reperfusion injury caused by reperfusion-related cardiac tissue damage and instigated arrhythmias [2, 22, 27, 28]. Furthermore, mitochondria are both a major energy and oxidative stress production site. DPP-4i can rescue cardiac mitochondrial dysfunction and decrease reactive oxygen species production in those patients with obesity-related insulin resistance and DM with ischemia/reperfusion injury. In addition, DPP4i can reduce oxidative stress [27, 29]. Diabetes is one of the comorbidities that can induce a systemic inflammatory state, while DPP-4i can reduce the systemic proinflammatory state and decrease oxidative stress, which may explain why DPP-4i decrease coronary microvascular endothelial inflammation and make a contribution to post-AMI remodeling during advanced heart failure [20]. Improvements in survival after AMI in T2 DM might also arise from modification of autophagy in the non-infarcted region of the heart [30]. Diabetes carries a two-fold increased risk of heart failure following myocardial infarction, due to an excessive loss of cardiac microvasculature. Stromal cell-derived factor-1alpha (SDF-1alpha) is a chemokine that is elaborated by ischemic tissue but is rapidly degraded by DPP-4i, thus, DPP-4i attenuates cardiac dysfunction and adverse remodeling in the post-MI setting [20].

Finally, DPP4i might inhibit atherosclerosis and proliferation of vascular smooth muscle cell. The possible impact of DPP-4i on post-AMI survival may derive from their cardioprotective effects and their beneficial effect on cardiovascular risk factors such as atherosclerosis and hypertension [4–8, 31–36]. In Japanese patients with diabetes and multiple CV risk factors, DPP4i was shown to decrease blood pressure associated with an improvement in albuminuria in addition to glycemic control [17]. In a sub-analysis of the PROLOGUE study, DPP-4i was shown not to alter endothelial function at the 2-year follow-up [37], a result which was further confirmed by another study [38].

### Benefit of DPP-4i-based combination therapy in AMI

Both DPP-4i and metformin have been shown to improve insulin resistance and attenuate myocardial injury caused by ischemia/reperfusion injury, and several studies have indicated that the combined therapy provided better outcomes than monotherapy with a reduction in arrhythmia scores and reduced all-cause mortality rates [39–43]. Previous studies revealed that both DPP-4i and metformin users had a significantly lower risk for composite cardiovascular diseases [18]. The combination therapy of DPP4i with conventional OHA led to an improvement in passive left ventricular compliance [20]. Interestingly, there were pleiotropic effects on cardiovascular protection using several OHAs, and their use was also associated with a lower risk of aortic aneurysm growth in Metformin-, sulfonylurea-, and TZD-treated patients but not in patients treated with DPP-4i or alpha-glucosidase inhibitors [19]. In our study, 1168 (43.71%) patients received both metformin and DPP-4i therapy, and the overall survival rates were better in those patients receiving DPP-4i. This benefit may be attributed to metformin (HR = 0.77; 95% CI 0.68–0.86) or DPP-4i (HR = 0.86; 95% CI 0.78–0.95). Furthermore, combined therapy with DPP-4i contributed to the improvement in survival rates independent of gender.

### Study limitations

Our study had several limitations. We did not use an objective indicator, such as glycated hemoglobin (HbA1c), as a standard for assessing diabetic compensation and treatment, due to bias in the frequency and interval of HbA1c check-ups. In addition, the DPP-4i group had a higher proportion of patients prescribed β-blockers, ARBs, spironolactone, and OHAs, which implied intractable hypertension, poorly controlled DM, or an unstable status. Regardless of the severity of diabetes or underlying conditions in the DPP-4i group, DPP-4i still made an independent contribution to the long-term survival of patients, which was also confirmed by Cox proportional hazard regression analysis.

### Strengths of this study

Previous studies analyzed relatively small samples, while our study enrolled 186,326 patients with first AMI, based on an entire population comprising 23,000,000 patients. Our large sample size reduced the variability in sampling statistics and also utilized a propensity score, one-to-one matching technique to minimize confounding factors between the DPP-4i and control groups. However, future prospective randomized studies are required to confirm our findings.

## Conclusions

This nationwide study showed that DPP-4i therapy improved the long-term survival of diabetic patients after first AMI, regardless of gender. Furthermore, DPP-4i therapy was shown to be especially beneficial in patients without peripheral vascular disease, ESRD, or COPD.

## Abbreviations

ACEI: angiotensin-converting enzyme inhibitor
ACS: acute coronary syndrome
AMI: acute myocardial infarction
ARB: angiotensin receptor blocker
CAROLINA: cardiovascular outcome study of linagliptin versus glimepiride in patients with type 2 diabetes
CCB: calcium channel blocker
CI: confidence interval
COPD: chronic obstructive pulmonary disease
CVA: cerebrovascular accident
DM: diabetes mellitus
DPP-4: dipeptidyl peptidase 4
DPP-4i: dipeptidyl peptidase 4 inhibitors
ESRD: end-stage renal disease
EXAMINE: cardiovascular outcomes study of alogliptin in subjects with type 2 diabetes and acute coronary syndrome
GLP-1: glucagon-like peptide 1
HbA1c: glycated hemoglobin A1c
HF: heart failure
HR: hazard ratio
IRB: Institutional Review Board
NHI: National Health Insurance
NHIRD: National Health Insurance Research Database
OHA: oral hypoglycemic agent
PCI: percutaneous coronary intervention
PKA: protein kinase A
PVD: peripheral vascular disease
SAVOR-TIMI: saxagliptin assessment of vascular outcomes recorded in patients with diabetes mellitus-thrombolysis in myocardial infarction
SD: standard deviation
SDF-1alpha: stromal cell-derived factor-1alpha
T2DM: type 2 DM
TECOS: Sitagliptin Cardiovascular Outcome Study
